# Supplementary material for: Deep learning model for fully automated breast cancer detection system from thermograms
Source: PLoS One. 2022 Jan 14;17(1):e0262349. doi: 10.1371/journal.pone.0262349 (PMC8759675; doi:10.1371/journal.pone.0262349)
Supplement: Attachment — Submitted filename: Response to Reviewers.docx [file pone.0262349.s002.docx]

**Original Manuscript ID:** PONE-D-21-28729

**Original Article Title: “**Deep Learning Model for Fully Automated Detection of Abnormal Breast Tissues from Thermograms “

**To:** PLOS ONE

**Re:** Response to reviewers

Dear Editor,

Thank you for allowing a resubmission of a revised version of the manuscript, with an opportunity to address the reviewers’ comments.

We are uploading (a) our point-by-point response to the comments (below) (response to reviewers), (b) an updated manuscript with yellow highlighting indicating changes, and (c) a clean updated manuscript without highlights

Best regards,

Authors,

**Reviewer#1, Concern # 1:** The existing literature should be classified and systematically reviewed, instead of being independently introduced one-by-one.

The literature is classified systematically as recommended

**Reviewer#1, Concern # 2:** The abstract is too general and not prepared objectively. It should briefly highlight the paper's novelty as what is the main problem, how has it been resolved and where the novelty lies?

The abstract is edited as recommended.

**Reviewer#1, Concern # 3:** The 'conclusions' are a key component of the paper. It should complement the 'abstract' and normally used by experts to value the paper's engineering content. In general, it should sum up the most important outcomes of the paper. It should simply provide critical facts and figures achieved in this paper for supporting the claims.

A conclusion section is added to the manuscript with your instructions.

**Reviewer#1, Concern # 4:** For better readability, the authors may expand the abbreviations at every first occurrence.

The manuscript is edited with abbreviation definition at every first occurrence.

**Reviewer#1, Concern # 5:** The author should provide only relevant information related to this paper and reserve more space for the proposed framework.

Information that is not related to this paper were removed. See the update in the Revised Manuscript with Track Changes page 16.

**Reviewer#1, Concern # 6:** However, the author should compare the proposed algorithm with other recent works or provide a discussion. Otherwise, it's hard for the reader to identify the novelty and contribution of this work.

A comparison between the proposed algorithm and other works is already exist in table 10. Also, we update this table with recent works. See the update in the Revised Manuscript with Track Changes page 18-19. From this table, we can note that the dataset used by our proposed system is large compared with the dataset of some related work. Also, our system extracts the breast area automatically by using U-Net network, but some related work doesn't used segmentation method and other extract it manually. In addition, the evaluation metric of our proposed system is better than related work. Furthermore, Statistical analysis by ANOVA test (see table 9) indicates the viability of the proposed system. In addition, the proposed system is domain-independent, so it has the ability to be applied to various computer vision tasks.

**Reviewer#1, Concern # 7:** The descriptions given in this proposed scheme are not sufficient that this manuscript only adopted a variety of existing methods to complete the experiment where there are no strong hypothesis and methodical theoretical arguments. Therefore, the reviewer considers that this paper needs more works.

To automate and improve the accuracy of thermography systems, we designed a deep learning-based system which integrates U-Net network and a proposed deep learning model. The proposed system is a combination two important methods: U-Net network and a two-class CNN-based deep learning model. First, U-Net is a convolutional network architecture which proved very strong in biomedical segmentation and very fast compared with other methods [29]. U-Net is used in our system to automatically extract and isolate the breast area from other parts of the body which act as noise in the detection system. Second, the two-class CNN-based deep learning model is trained from scratch to extract more characteristics from the dataset that is helpful in training the network and improve the efficiency of the classification process. The novelty of the proposed system lays in using U-Net network for automating the segmentation process and building a deep learning model which use the output of U-Net to classify the given thermogram. The combination between U-Net and our proposed deep learning model proved to be effective as it achieved accuracy= 99.33%, sensitivity=100% and specificity=98.67%

**Reviewer#1, Concern # 8:**  Key contribution and novelty has not been detailed in manuscript. Please include it in the introduction section.
We add the main contribution of this paper in the introduction section. To quickly and easily check this update, it is in page 4 of Revised Manuscript with Track Changes, it is also copied below:

The main contribution of this paper is as following:

1-Extracting and isolating the breast area automatically from other parts of thermal images by using CNN (U-Net).

2- Proposing a deep learning model for the classification of normal and abnormal breast tissues from thermograms

3-Evaluating the performance of the proposed model using accuracy, sensitivity and specificity.

4-Comparing the proposed model with state-of art methods.

**Reviewer#1, Concern # 9:**  What are the limitations of the related works?

Limitations of the related work are added in the literature review section.

**Reviewer#1, Concern # 10:** Are there any limitations of this carried out study?

1- The computation time of the segmentation process is high due to the limitation of the PC capabilities used in this study.
2- The proposed deep learning model for classification has a bad behavior when the learning rate is greater than 3.0 *e^−^*^03^

**Reviewer#1, Concern # 11:** How to select and optimize the user-defined parameters in the proposed model?

There is no general rule for it. Selecting the optimum number of the user-defined parameters is dependent on the performance of the model and the result of the evaluation metrics.

**Reviewer#1, Concern # 12:** There are quite a few abbreviations are used in the manuscript. It is suggested to use a table to host all the frequently used abbreviations with their descriptions to improve the readability

Thank you for your comment. We add a table of abbreviation to the manuscript. To quickly and easily check this table, it is copied below:

| **Abbreviation** | **Definition** |
| --- | --- |
| **CNN** | Convolutional Neural Networks |
| **CAD** | Computer-Aided Detection |
| **ROI** | Region of Interest |
| **EHMM** | Extended Hidden Markov Models |
| **NS** | Neutrosophic Sets |
| **F-FCM** | Fast Fuzzy C-Mean |
| **SVM** | Support Vector Machine |
| **KNN** | K-Nearest Neighbor |
| **NN** | Naïve Bayes |
| **PCA** | Principal Component Analysis |
| **DNN** | Deep Neural Network |
| **DWNN** | Deep-Wavelet Neural Networks |
| **RELU** | Rectified Linear Activation Function |
| **ADAM** | Adaptive Moment Estimation |
| **SGDM** | Stochastic Gradient Descent with Momentum |
| **RMSprop** | Root Mean Square propagation |
| **T_P_** | True Positive |
| **T_N_** | True Negative |
| **F_P_** | False Positive |
| **F_N_** | False Negative |
| **GLCM** | Gray Level Co-occurrence Matrices |
| **HOG** | Histogram of Oriented Gradients |
| **n** | normal |
| **ab** | abnormal |

**Reviewer#1, Concern # 13:** Explain the evaluation metrics and justify why those evaluation metrics are used?

We edit evaluation the deep learning model section as following. To quickly and easily check this update in the Revised Manuscript with Track Changes page 11. Also, it is copied below:

Classification Metrics evaluate the performance of the model and measure how good or bad the classification is.

**Accuracy:** represents how many instances are completely classified correctly. It is calculated by dividing the total number of predictions by the number of right predictions**.** It is calculated by Eq. (3)

$\boldsymbol{Accuracy}\mathbf{=}\frac{\boldsymbol{T}_{\boldsymbol{P}}\mathbf{+}\boldsymbol{T}_{\boldsymbol{N}}}{\boldsymbol{T}_{\boldsymbol{P}}\mathbf{+}\boldsymbol{T}_{\boldsymbol{N}}\mathbf{+}\boldsymbol{F}_{\boldsymbol{P}}\mathbf{+}\boldsymbol{F}_{\boldsymbol{N}}}$	(3)

**Sensitivity:** is calculated based on how many patients have the disease are correctly estimated. It is calculated by Eq. (4)

$\boldsymbol{Sensitivity=}\frac{\boldsymbol{T}_{\boldsymbol{P}}}{\boldsymbol{T}_{\boldsymbol{P}}\boldsymbol{+}\boldsymbol{F}_{\boldsymbol{N}}}$	(4)

**Specificity:** is calculated based on how many patients do not have the disease are predicted right. It is calculated by Eq. (5)

$\boldsymbol{Specificity=}\frac{\boldsymbol{T}_{\boldsymbol{N}}}{\boldsymbol{T}_{\boldsymbol{N}}\boldsymbol{+}\boldsymbol{F}_{\boldsymbol{P}}}$	(5)

•*True Positive* (TP) refers to a positive-class sample that has been successfully classified by a model.

• *False Positive* (FP) refers to a sample that should have been classed as negative but was instead classified as positive.

• *True Negative* (TN) refers to a negative-class sample that has been successfully classified by a model.

• *False Negative* (FN) refers to a sample that should have been classed as positive but was instead classified as negative.

The accuracy metric indicates how many of the model's predictions were right. However, if the dataset is unbalanced, a model's high accuracy rate does not guarantee its ability to differentiate distinct classes equally. In specifically, in the classification of medical images, it is necessary to develop a model with the ability be applied to all classes. In cases, sensitivity and specificity should be used to provide information about the performance of the model. Sensitivity measures the percentage of patient have the disease that the proposed model correctly predicted. Specificity measures the percentage of patient do not have the disease and correctly estimated by the proposed model. These two evaluation metrics measure the ability of the model to decrease *FN* and *FP* predictions.

**Reviewer#1, Concern # 14:** Some sentences are too long to follow, it is suggested that to break them down into short but meaningful ones to make the manuscript readable

We try to break some sentences down into short as much as we can.

**Reviewer#1, Concern # 15:** The title is pretty deceptive and does not address the problem completely.

The title of the article is changed into " Deep Learning Model for Fully Automated Breast Cancer Detection System from Thermograms"

**Reviewer#1, Concern # 16:** Every time a method/formula is used for something, it needs to be justified by either (a) prior work showing the superiority of this method, or (b) by your experiments showing its advantage over prior work methods - comparison is needed, or (c) formal proof of optimality. Please consider more prior works.

The limitations of the prior work are added to the literature review section. To quickly and easily check update in the Revised Manuscript with Track Changes page 7. Also, it is copied below:

From the discussed related work above, it could be remarked that the prior work has some limitations such as:

(1) some related work used a small number of the dataset as in [6, 20].

(2) some related work did not consider segmentation of the breast area before classification such as in [27] or extract the breast area manually such as in [20, 21].

(3) some segmentation models such as in [1] removed parts of the breast.

(4) some work has been evaluated by only calculating the accuracy metric only such as in [29]. However, the high accuracy rate of a model does not ensure its ability to distinguish different classes equally if the dataset is unbalanced [39].

Therefore, a fully automated breast cancer detection system from thermograms is needed and should be evaluated by not only the accuracy but also the most related metrics such as sensitivity and specificity.

Also, more recent prior works are added to the literature review section and compared with the proposed method in table 10.

**Reviewer#1, Concern # 17:** The data is not described. Proper data description should contain the number of data items, number of parameters, distribution analysis of parameters, and of the target parameter itself for classification.

A data description table is added to the manuscript as table1. To quickly and easily check this table, it is copied below:

| Dataset categories | Dimension | Training | Validation | Testing | Total |
| --- | --- | --- | --- | --- | --- |
| Normal | 640x480 | 350 | 75 | 75 | 500 |
| Abnormal | 640x480 | 350 | 75 | 75 | 500 |

**Reviewer#1, Concern # 18:** The related works section is very short and no benefits from it. I suggest increasing the number of studies and add a new discussion there to show the advantage. Following studies can be considered
a. Breast-Cancer Detection using Thermal Images with Marine-Predators-Algorithm Selected Features.
b. Dilated Semantic Segmentation for Breast Ultrasonic Lesion Detection Using Parallel Feature Fusion.
c. Machine-Learning-Scheme to Detect Choroidal-Neovascularization in Retinal OCT Image

The recommended studies are cited in the manuscript as following:

a🡪23- Rajinikanth V, Kadry S, Taniar D, Damaševičius R and Rauf H T. Breast-Cancer Detection using Thermal Images with Marine-Predators-Algorithm Selected Features. In: 2021 Seventh International conference on Bio Signals, Images, and Instrumentation (ICBSII); Chennai, India. 2021.

b🡪30- Irfan R, Almazroi A, Rauf H, Damaševiˇcius R, Nasr E and Abdelgawad A. Dilated Semantic Segmentation for Breast UltrasonicLesion Detection Using Parallel Feature Fusion. Diagnostics*.*2021; 11: 1212.

c🡪 36- Rajinikanth V, Kadry S, Damaševičius R, Taniar D and Rauf H T. Machine-Learning-Scheme to Detect Choroidal-Neovascularization in Retinal OCT Image. In: 2021 Seventh International conference on Bio Signals, Images, and Instrumentation (ICBSII); Chennai, India. 2021.

**Reviewer#1, Concern #19:** Use Anova test to record the significant difference between performance of the proposed and existing methods.

We add statistical analysis section to the manuscript. To quickly and easily check this section in the Revised Manuscript with Track Changes page 16. Also, it is copied below:

**4.7 Statistical Analysis**

To analyze the evaluation of the proposed system statistically, we perform the analysis of variance (ANOVA) test, where the proposed system is compared with ResNet18, GoogleNet and VGG16 networks. The result of the ANOVA test is shown in table 9. To reject the null hypothesis, the *p-value* in the ANOVA test should be less than 0.05. According to Table 9, the *p*−*value* is less than 0.05. So, the null hypothesis was rejected by the results of ANOVA test.

TABLE 9. **Results of the ANOVA test of the proposed model and CNN models**

| **Model** | **P-value** |
| --- | --- |
| ResNet18 | 0.0423 |
| GoogleNet | 0.0173 |
| VGG16 | 0.0023 |

**Reviewer#2, Concern # 1:** The image size is fixed as 228x228, but, the initial input dimension in UNet is depicted as 572x572. Please check and correct.

Thank you for comment. But, if you define the network, then you can change the input dimension in the input layer to your desired one.

**Reviewer#2, Concern # 2:** The quality of the image is to be improved in every image cases.

Thank you for your comment. The quality of the image is improved

**Reviewer#2, Concern # 3**: What is the need for extracting the breast section, please justify. Further, include, how the CNN scheme is trained to segment the image (a clear discription is needed).

*We add the answer of this question in* ***Breast area segmentation using deep learning (CNN)*** *section.* To quickly and easily check this section in the Revised Manuscript with Track Changes page 8. Also, it is copied below:

The thermal image contains unnecessary areas as neck, shoulder, chess and other parts of the body which acts as noise during the training in CNN models. The aim of extracting the breast section is removing unwanted regions and using the areas destined to have cancer as the input of the CNN model for training and testing.

In U-Net, the initial series of convolutional layers are combined with max pooling layers to decrease the resolution of the input image. Then, these layers are followed by a series of convolutional layers combined with upsampling operators in order, so the resolution of the input image is increased. Combining these two paths produces a U-shaped graph that can used to perform image segmentation. For breast area segmentation with U-Net, Adaptive Moment Estimation (ADAM) method was used as optimized algorithm with number of epochs = 30. In addition, the training process was started with initial learning rate = 1.0e−3. The learning rate used a piecewise schedule and dropped by a factor of 0.3 every 10 epochs to allow the network to train quickly with a higher initial learning rate. The network trained with an 8-batch size to save memory.

**Reviewer#2, Concern # 4:** The image dimension, such as test image, Ground truth and segmented image is not looks like 228x228. Further in this database, the Ground Truth is not available. Please discuss, how the GT is generated (Hope, the test images are from:http://visual.ic.uff.br/dmi/).

Thank you for your comment. All images are resized to 228x228. Ground Truth images is not available for general in the site, we get it by personal communication. The test images are from the site.

**Reviewer#2, Concern # 5** Please improve the reference section by considering few more related works.(Please refer, the following research works:
a. Breast-Cancer Detection using Thermal Images with Marine-Predators-Algorithm Selected Features
b. An Examination System to Classify the Breast Thermal Images into Early/Acute DCIS Class
c. A hybrid framework to evaluate breast abnormality using infrared thermal images

The recommended studies are cited in the manuscript as following:

a->23- Rajinikanth V, Kadry S, Taniar D, Damaševičius R and Rauf H T. Breast-Cancer Detection using Thermal Images with Marine-Predators-Algorithm Selected Features. In: 2021 Seventh International conference on Bio Signals, Images, and Instrumentation (ICBSII); Chennai, India. 2021.

b->20- Dey N, Rajinikanth V and Hassanien A. E. An Examination System to Classify the Breast Thermal Images into Early/Acute DCIS Class. In: Proceedings of International Conference on Data Science and Applications; Singapore. 2021.

c->10- Fernandes S, Kadry S and Rajinikanth V. A hybrid framework to evaluate breast abnormality using infrared thermal images. IEEE Consumer Electronics Magazine*.* 2019; 8: 31-36.

**Reviewer#2, Concern # 6** I request you to compare the outcome of the proposed scheme with another results existing in the literature.

Thank you for your comment. This comparison is already existing in Table 10. Also, we compare the proposed scheme with the related work considered in comment 5 in the same table.
