# Supplementary material for: Deep learning model for fully automated breast cancer detection system from thermograms
Source: PLoS One. 2022 Jan 14;17(1):e0262349. doi: 10.1371/journal.pone.0262349 (PMC8759675; doi:10.1371/journal.pone.0262349)
Supplement: Attachment — Submitted filename: Response to Reviewers.docx [file pone.0262349.s003.docx]

**Original Manuscript ID:** PONE-D-21-28729R1

**Original Article Title: “**Deep Learning Model for Fully Automated Breast Cancer Detection System from Thermograms “

**To:** PLOS ONE

**Re:** Response to reviewers

Dear Editor,

Thank you for allowing a resubmission of a revised version of the manuscript, with an opportunity to address the reviewers’ comments.

We are uploading (a) our point-by-point response to the comments (below) (response to reviewers), (b) an updated manuscript with yellow highlighting indicating changes, and (c) a clean updated manuscript without highlights

Best regards,

Authors,

**Improve the quality of all figures before submitting final version.**

Thank you for your comment. The quality of figures is improved

**Improve Fig. 10. The data is not discernable. Range Y-axis to [80,100]. Add the units of measurement (percentages).**

Thank you for your comment. Figure 10 is improved as per your instruction.

**Include the data availability statement and the acknowledgement for "http://visual.ic.uff.br/dmi/".**

Thank you for your comment. The acknowledgement for ([*http://visual.ic.uff.br/dmi*](http://visual.ic.uff.br/dmi)) is added to the paper. To quickly and easily check this, it is in page 20 of Revised Manuscript with Track Changes, it is also copied below:

Acknowledgement

The authors would like to thank the Department of Computer Science and the Hospital of the Federal University Fluminense, Niterói, Brazil, for providing DMR-IR benchmark database which is accessible through an online user-friendly interface ([*http://visual.ic.uff.br/dmi*](http://visual.ic.uff.br/dmi)) and used for experiments.
